# Isolation and Identification of Mercury-Tolerant Bacteria LBA119 from Molybdenum-Lead Mining Soils and Their Removal of Hg^2+^

**DOI:** 10.3390/toxics11030261

**Published:** 2023-03-12

**Authors:** Hanyue Yao, Hui Wang, Jiangtao Ji, Aobo Tan, Yang Song, Zhi Chen

**Affiliations:** 1 School of Chemical Engineering and Pharmacy, Henan University of Science and Technology, Luoyang 471023, China; 2 College of Agricultural Equipment Engineering, Henan University of Science and Technology, Luoyang 471023, China; 3 Department of Building, Civil and Environmental, Concordia University, Montreal, QC H3G 1M8, Canada

**Keywords:** *Bacillus*, 16S rDNA, heavy metal mercury, soil

## Abstract

Aims: To screen heavy metal-tolerant strains from heavy metal-contaminated soil in mining areas and determine the tolerance of the strains to different heavy metals and their removal rates through experiments. Methods: Mercury-resistant strain LBA119 was isolated from mercury-contaminated soil samples in Luanchuan County, Henan Province, China. The strain was identified by Gram staining, physiological and biochemical tests, and 16S rDNA sequences. The LBA119 strain showed good resistance and removal rates to heavy metals such as Pb^2+^, Hg^2+^, Mn^2+^, Zn^2+^, and Cd^2+^ using tolerance tests under optimal growth conditions. The mercury-resistant strain LBA119 was applied to mercury-contaminated soil to determine the ability of the strain to remove mercury from the soil compared to mercury-contaminated soil without bacterial biomass. Results: Mercury-resistant strain LBA119 is a Gram-positive bacterium that appears as a short rod under scanning electron microscopy, with a single bacterium measuring approximately 0.8 × 1.3 μm. The strain was identified as a *Bacillus* by Gram staining, physiological and biochemical tests, and 16S rDNA sequence analysis. The strain was highly resistant to mercury, with a minimum inhibitory concentration (MIC) of 32 mg/L for mercury. Under a 10 mg/L mercury environment, the optimal inoculation amount, pH, temperature, and salt concentration of the LBA119 strain were 2%, 7, 30 °C, and 20 g/L, respectively. In the 10 mg/L Hg^2+^ LB medium, the total removal rate, volatilization rate, and adsorption rate at 36 h were 97.32%, 89.08%, and 8.24%, respectively. According to tolerance tests, the strain showed good resistance to Pb^2+^, Mn^2+^, Zn^2+^, Cd^2+^, and other heavy metals. When the initial mercury concentration was 50 mg/L and 100 mg/L, compared with the mercury-contaminated soil that contained an LB medium without bacterial biomass, LBA119 inoculation increased 15.54–37.67% after 30 days of culture. Conclusion: This strain shows high bioremediation potential for mercury-contaminated soil.

## 1. Introduction

Mercury pollution in water bodies due to human activities is mainly caused by wastewater from industries that produce items such as chlor-alkali, plastics, batteries, electronics, and used medical devices [1]. It is estimated that approximately 3.2 × 10^4^ hm of agricultural land in China is contaminated with mercury. Mercury is highly toxic and can cause severe damage to human cells and limit normal cellular function when it enters the human body [2]. Mercury is distributed in the atmosphere, soil, and water bodies; both soluble and insoluble mercury compounds are partially volatilized into the atmosphere, and the transport transformation of mercury occurs between land, water, and air. A general rule of thumb is that, compared to inorganic mercury, organic mercury is more volatile [1], and methylmercury is the most volatile. Through microorganisms, inorganic mercury can be converted into more toxic methylmercury, dimethylmercury [3], etc.

Numerous studies have examined the possibility of removing mercury with microbial organisms. For example, Yang Wen screened a mercury-resistant strain in a mercury-rich soil upstream of Miyun Reservoir in Beijing, and this strain was identified as *Pseudomonas aeruginosa* [4]. When mercury concentrations were 10 mg/L for 24 h under optimum conditions, the rates of mercury removal were over 90%. Zheng Yan et al. reported a highly resistant strain of mercury, *Pseudomonas aeruginosa*, which can rapidly volatilize more than 68% of Hg^0^ within 8 h, and Fan Taotao et al. isolated three strains of mercury-resistant and mercury-reducing bacteria from mercury-contaminated water in a chemical plant [5], and these strains exhibit a mercury removal rate of more than 90% under optimal conditions. The mercury-tolerant strains in this study were selected from agricultural fields that were contaminated with heavy metals near molybdenum mines in Luanchuan County, and the rate of mercury removal was approximately 96% at mercury concentrations of 2–12 mg/L, a higher percentage than that previously reported in the literature.

Heavy metal-contaminated soil located around mining areas is a valuable source for isolating strains tolerant to heavy metals [6]. A strain with mercury tolerance was isolated from agricultural fields in the molybdenum mining area of Luanchuan County, Luoyang. Its biological properties and volatile mercury energy were initially investigated. The possibility of using this strain to remediate mercury-contaminated environments was evaluated to provide a reference for future microbial remediation of mercury-contaminated soils in mining areas.

## 2. Materials and Methods

### 2.1. Soil Sample Source

In this experiment, mercury-contaminated soils were collected from agricultural fields in Luanchuan County, Luoyang City, Henan Province (33° N, 111° E) using the five-point sampling method, and the samples were primarily from the protoplasmic soil layer within the top 20 cm of the soil. The mercury content of the soil was determined by atomic fluorescence with a detection limit of 0.002 mg/kg.

### 2.2. Screening Mercury-Resistant Strains

Soil samples were collected from the heavy metal-contaminated soil in the mining area; 10 g of soil was placed in a 250 mL beaker, 90 mL of sterile water was added to create a bacterial suspension and the suspension was magnetically stirred for 30 min and left to stand. Then, 1 mL of supernatant was inoculated with a pipette gun in an LB medium containing 10 mg/L Hg^2+^ in a 30 °C; the samples were shaken at 150 r/min in an incubator and the samples were incubated for 2 h for 10× series (10^−2^, 10^−3^, 10^−4^ and 10^−5^) gradient dilutions [1]. A dilution of the sample was spread on a solid LB medium containing 20 mg/L Hg^2+^ and incubated in a biochemical incubator at 37 °C for 48 h. After the colonies grew on the medium, the concentration of mercuric chloride in the LB solid medium was increased continuously. A high concentration of mercury-tolerant strains was screened by picking and culturing individual colonies on an LB solid medium containing a high mercury concentration.

The selected mercury-resistant strains were cultured in line on LB solid medium without mercury, and the strains were repeatedly purified and screened. The morphological characteristics of the colonies on the medium were observed and recorded. Inoculations of the mercury-resistant strain were placed into LB liquid medium and were incubated at 30 °C and 150 rpm in a constant temperature shaker until the solution became cloudy. The solution was preserved using glycerol. With the aid of a pipette on an ultraclean table, 0.2 mL of glycerol was pipetted into a 1.5 mL centrifuge tube, and 0.2 mL of the bacterial seed solution was mixed with gentle shaking. The 1.5 mL centrifuge tube containing the bacterial solution was placed in a sealed bag and placed in a refrigerator at −40 °C for use in subsequent experiments.

### 2.3. Strain Identification

The bacteria were identified by colony morphology and biochemical tests. Biochemical tests included Gram stain [7], the starch hydrolysis test, gelatin hydrolysis test, oil hydrolysis test, indole test, sugar fermentation test, methyl red test, volt-pop (VP) test, H_2_S test, and the strain motility test.

### 2.4. Molecular Biology Identification

Phylogenetic trees are used to represent the affinities between various organisms and to infer the evolutionary history of species through the study of biological sequences, mainly through DNA sequences, protein sequences, protein structures, etc. Phylogenetic trees are constructed. The phylogenetic tree in this study was constructed based on the 16S rDNA of LBA119 bacteria and was compared with the validated 16S rDNA in the NCBI library.

Genomic DNA of the strain was extracted by the boiling template method, and the 16S rDNA gene [8] was amplified by polymerase chain reaction (PCR) using bacterial universal primers 27F (5′-AGAGTTTGATCMTGGCTCAG-3′) and 1492R (5′-ACGGCTACCTTGTTACGA-3′). The temperature program was to preheat at 95 °C for 5 min, denaturation at 95 °C for 1 min, 55 °C annealing for 1 min, and 72 °C extension for 2 min, repeated for 30 cycles, and finally 72 °C extension for 10 min. The composition of the PCR reaction system is shown in Table 1. The PCR [9] products were sent to Shanghai Bioengineering Co., Ltd (Sangon Biotech (Shanghai) Co., Ltd., Shanghai, China). for sequencing, and the sequencing results were compared with the GenBank database of the National Center for Biotechnology Information (NCBI) to identify the genus of the strain. The phylogenetic tree was constructed by the neighbor-joining method (distance-based approach) of the MEGA X software (Version 11.0.13).

### 2.5. Exploration of Optimal Growth Conditions for Bacteria Strains

To investigate the effect of different media on the growth of experimental strains [10], 50 mL of each of eight liquid media, including an LB medium, beef paste peptone medium, high type 1 medium, PDA medium, Choi medium, sand medium, glycerol medium, and PSA medium, was prepared in a 250 mL conical flask, and a standard liquid medium containing 10 mg/L Hg^2+^ and no Hg^2+^ was prepared. Inoculation was performed after the medium was adjusted to pH 7.0, sterilized at 121 °C for 30 min, and cooled (fresh bacterial seed solution to medium = 2:100). The culture was incubated for 36 h at 37 °C and 150 r/min in a constant temperature shaking incubator. The cultured bacterial solution was centrifuged at 8000 rpm for 10 min, the supernatant was discarded, and the centrifuge tube was placed in a vacuum drying oven and dried at 70 °C to a constant weight. Each group was divided into three replicates. The dry weight of the strain was calculated as follows:

$$m=\left(M_{1}-m_{1}\right)-\left(M_{2}-m_{2}\right)$$

where M_1_ is the initial mass of the experimental group, M_2_ is the initial mass of the control group, m_1_ is the mass of the experimental group after drying, and m_2_ is the mass of the control group after drying.

Under the optimum conditions, LB liquid medium containing 10 mg/L Hg^2+^ and LB liquid medium without Hg^2+^ were used. The following range of inoculums was used for the test strains: 1%, 2%, 3%, 4%, 5%, 6%, 7%, and 8%, and the OD_600nm_ values were determined after 36 h of incubation at 150 rpm and 30 °C.

The pH of the liquid LB medium was adjusted to 4, 5, 6, 7, 8, 9, and 10 with hydrochloric acid (HCl) or sodium hydroxide (NaOH), and the OD_600nm_ values were measured after inoculating fresh bacterial solutions into LB liquid medium and incubating the solutions at 37 °C for 36 h at 150 rpm to obtain the proper pH range for strain growth.

Based on the same medium preparation method, pH, and inoculation method, the OD600 nm values were determined after 36 h of incubation [11] at 20 °C, 25, 30, 35 °C, and 40 °C in a constant temperature shaking incubator. Under the above optimal conditions, the bacteria were inoculated in liquid LB [12] media containing 0, 0.5%, 1%, 2%, 4%, 6%, and 8% sodium chloride, and the OD_600 nm_ values were measured after incubation to obtain the optimal salt concentration. Each group of experiments was repeated three times.

### 2.6. Determination of the Tolerance of Strains to Mercury

Under optimal growth conditions, LB liquid medium was prepared with Hg^2+^ concentrations of 0, 1, 2, 4, 8, 16, 32, 64, and 128 mg/L, sterilized at 121 °C for 30 min, inoculated with 2% fresh bacterial broth into LB liquid medium, and incubated with shaking for 120 h. To determine the minimum inhibitory concentration of heavy metal mercury on the experimental strains, the optical density (OD_600nm)_ values of each medium were measured regularly. The minimum inhibitory concentration (MIC) of Hg^2+^ in the experimental strains was determined at regular intervals by measuring the OD_600 nm_ values of each medium for 120 h.

According to the minimum inhibitory concentration (MIC) of Hg^2+^ on the strains, 2% fresh bacterial solution was inoculated into the liquid LB medium with 5, 10, and 20 mg/L Hg^2+^; an LB medium without mercury was used as a blank control, and the optical density was measured regularly in a UV spectrophotometer to study the changes in the growth pattern of the strains under mercury stress.

### 2.7. Determination of Mercury Volatilization and Adsorption Capacity of the Strain

As part of the experiment, three groups were established. The positive control group contained no mercury, the negative control group was not inoculated with the strain. The experimental group was inoculated with 2% fresh bacterial broth in a liquid LB medium containing 10 mg/L Hg^2+^ and under optimal growth conditions, and three parallel treatments were conducted for each group. The samples were removed at regular intervals and centrifuged at 8000 rpm for 10 min. The process of digesting the supernatant and bacterium was performed as follows. A total of 1 mL supernatant from the centrifuged culture solution was mixed with 10 mL of HCl in a 50 mL Teflon crucible, the sample was heated continuously on an electric furnace, and the sample was digested until the volume was reduced to 3 mL. A total of 5 mL nitric acid was added to the solution, and the solution was heated for 1 h. The steps were repeated until the digestion was complete and the white fumes were exhausted. Then, the digestion solution was transferred to a 50 mL volumetric flask after it had cooled to room temperature and was shaken well with a fixed volume.

A cold atomic absorption spectrophotometer was used to measure the mercury concentration in the supernatant and the mercury content in the bacteria. The efficiency of total mercury removal was the initial mercury content of the culture medium minus the mercury content of the supernatant, divided by the initial mercury content of the culture medium; the efficiency of mercury adsorption by the bacteria was determined by the mercury content of the bacteria divided by the initial mercury content of the culture medium.

The mercury concentration of the supernatant and the mercury content of the bacteria were measured using a cold atomic absorption spectrophotometer. The efficiency of total mercury removal is the initial mercury content of the culture medium minus the mercury content of the supernatant, divided by the initial mercury content of the culture medium, and the efficiency of mercury adsorption by the bacteria is the mercury content of the bacteria divided by the initial mercury content of the culture medium. The total removal rate of mercury minus the adsorption efficiency of the bacteria on mercury is the volatilization efficiency of mercury [13]. The heavy metal concentrations in the digestion solution and the blank control were measured three times. The calculation was performed as follows:

$$q_{1}=\frac{\left(a_{1}-b_{1}\right)}{a_{1}}$$

$$q_{2}=\frac{c_{1}}{a_{1}}$$

$$q_{3}=q_{1}-q_{2}$$

where a_1_ is the initial mercury content of the culture solution (mg/L); b_1_ is the mercury content of the supernatant (mg/L); c_1_ is the mercury content of the bacteria (mg/L); q_1_ is the total removal rate (%); q_2_ is the adsorption efficiency (%); and q_3_ is the volatilization efficiency (%).

### 2.8. Transformation Infrared Spectroscopy

To investigate the response mechanism of the LBA119 strain to mercury stress, Fourier transform infrared (FTIR) spectroscopy [11] was used to analyze the changes in the adsorption functional groups of bacteria before and after treatment with the heavy metal Hg^2+^. The bacteria were incubated in an LB medium with mercury (10 mg/L) and without mercury for 36 h. The cultures were centrifuged at 8000 rpm for 10 min, the supernatant was discarded, and the organisms were washed three times with phosphate buffer solution. The strains were freeze-dried under a vacuum, and the dried samples were thoroughly mixed with potassium bromide (KBr) powder in an agate mortar (bacterial mass: KBr mass = 1:100), pressed into thin slices using a solid press, and measured using a Fourier infrared spectrometer to record the infrared spectra in the region of 4000–400 cm^−1^. The obtained spectral data were processed using Omnic 9.0 software (OMNIC 8.2.0.387).

### 2.9. Testing the Strains for Resistance to Different Types of Heavy Metal Ions

LB liquid medium containing single heavy metal ions (Mn^2+^, Zn^2+^, Pb^2+^, Cd^2+^, and Cr^6+^) was prepared and inoculated with 2% fresh bacterial broth and incubated on a shaker at 30 °C for approximately 36 h. An LB medium with different concentration gradients (1, 100, 500, 1000, 1500, 2000, 3000, 4000, and 5000 mg/L) of heavy metals was first set to determine the crude tolerance of the strain to heavy metal concentrations [14]. The concentration interval was then narrowed to determine the maximum concentration of heavy metals that could be tolerated. Bacterial concentrations were determined using the 600 nm optical density method (OD_600nm_) to determine the minimum inhibitory concentration (MIC). Five heavy metal resistance assays were performed independently [15], with three parallel groups for each group.

### 2.10. Study on the Bioremediation Effect of Strains of Bacteria on Mercury-Contaminated Soil

In this experiment, mercury-contaminated soil was collected in Luanchuan County, Henan Province, from farmland in the molybdenum mining area. The soil was air-dried and sieved, and then a specific quantity of HgCl_2_ solution was added to it. The soil samples were prepared with mercury concentrations of 5, 50, and 100 mg/L, stirred well, and left to stand for several days before being stirred again; then, the mercury soil was aged for 60 days.

### 2.11. Strain for Remediation of Mercury-Contaminated Soil

A logarithmic growth stage was achieved by incubating the bacteria. An adjustment of pH 4.6 was made to the seed solution used for inoculating LBA119 bacteria. A blank LB medium was added to the control group at the same concentration. After inoculation, the soil and inoculum were gently mixed in a shaker at 30 rpm for 20 min to conduct an exploratory experiment on the biological removal of mercury from the soil [16].

The study involved several experimental groups, which were prepared as follows. (1) Restoration group: 100 g of mercury-aged soil samples, 95 mL of distilled water, and 5 mL of bacterial seed solution were added and shaken well to ensure that all the soil was suspended in the liquid. Sterile distilled water was replenished quantitatively every day to maintain the water content of the soil, and the new bacterial solution was added every 7 days, with an additional amount of 5 mL each time. (2) Control group: 100 g of aged soil samples containing mercury, 95 mL of distilled water, and 5 mL of the LB medium were added and shaken well, and 5 mL of fresh medium was added every 7 days.

In each experimental group, three replicates were conducted. Incubation for 7, 15, and 30 days was conducted in an incubator (temperature 30 °C, 8 h of light time and 16 h of darkness (simulated mine light time)). Each group was provided 0.5 g of air-dried soil that was ground and digested. Using cold atomic fluorescence spectrometry [17], the mercury content was determined, and the mercury removal efficiency was calculated.

Mercury remediation efficiency was calculated by subtracting the initial mercury content of the soil from the mercury content at the time of sampling. The results are the mean ± standard deviation of three independent replicates. The efficiency of the strains for remediating mercury in the soil was calculated as follows:

$$q\left(\%\right)=\frac{a-b}{a}\times 100\%$$

where q is the removal efficiency (%) of mercury in soil by the strain; a is the initial mercury content of soil; and b is the mercury content of soil at different sampling times.

## 3. Results

### 3.1. Determination of Soil Mercury Content

According to the results of the atomic fluorescence spectrometry analysis, the mean mercury concentration in the soil was 10.46 mg/kg, which is considerably higher than the background value for Chinese soil in the environment.

### 3.2. Screening and Purification of Mercury-Resistant Strains

**Figure 1:**
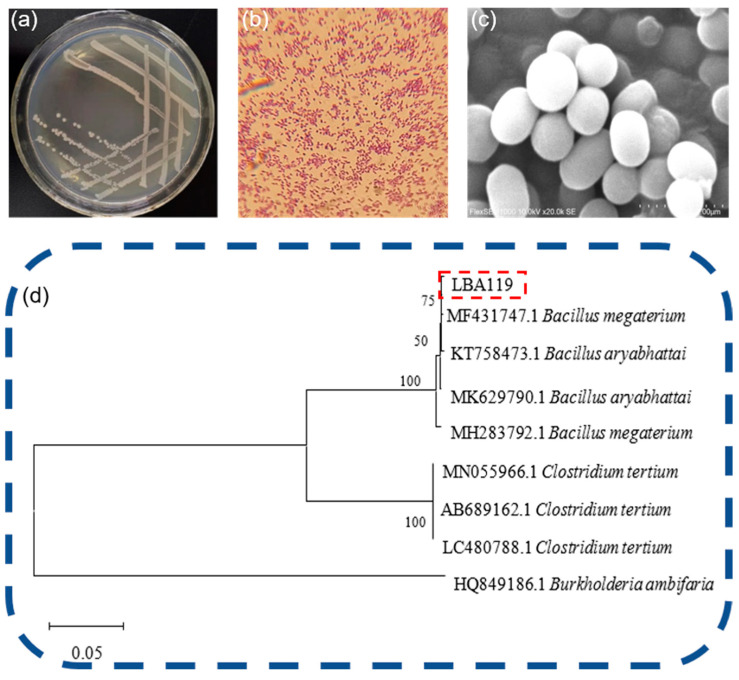
(**a**) LBA119 morphological growth diagram; (**b**) LBA119 Gram stain; (**c**) LBA119 electron microscope scan; (**d**) LBA119 phylogenetic tree.

The bacterial suspension was diluted in LB solid plates containing 20 mg/L Hg^2+^ and was incubated at 37 °C for 48 h in a biochemical incubator to obtain mercury-resistant colonies. The mercury-resistant strains with a positive growth status were selected from the plates, numbered LBA119, and enriched after multiple scribing to separate individual colonies. The enriched strains were preserved in glycerol. The LBA119 colonies demonstrated the morphological characteristics shown in Figure 1a after incubation at 37 °C for 24 h under constant temperature and inversion. Specifically, the colony size was 0.5–1 mm; the bacteria exhibited a smooth edge, round shape, smooth surface, consistent uniformity, no halo, a creamy white color with a light yellow. The bacteria were not sticky, easy to pick, highly flat and produced a rotten egg smell. By Gram staining, these bacteria were observed as Gram-positive bacteria under the biological microscope (Figure 1b). Scanning electron microscopy was used to observe the morphology of the bacterium: the LBA119 bacteria were fixed in 1% glutaraldehyde solution for 20 min and then centrifuged, rinsed and centrifuged several times using phosphate buffer, then dehydrated (3–4 times) with increasing concentrations of ethanol (30%, 50%, 70%, 80%, 90%, 100%) step by step, and finally the ethanol solution containing bacteria was dropped on clean polished aluminum sheets, dried, sprayed with gold, and microscopic images of bacterial cells were observed with scanning electron microscopy. The size of individual organisms was approximately 0.8 × 1.3 μm [18] under electron microscopy (Figure 1c).

### 3.3. Identification of Mercury-Resistant Strains

The bacterial isolates were identified according to biochemical characteristics and the 16S rDNA gene [19]. Referring to Table 2, the experimental results showed that strain LBA119 was positive for starch hydrolysis, gelatin hydrolysis, glucose fermentation, lactose fermentation, indole assay, methyl red, citrate utilization assay, and motility assay. It was negative for oil hydrolysis, VP, and H_2_S. The phylogenetic tree of the strain is shown in Figure 1d, and the 16S rDNA gene sequence comparison showed that the strain was 99% similar to strain LBA119 and strain KT758473.1 *Bacillus* aryabhattai. The strain LBA119 was identified as *Bacillus* [20].

### 3.4. Optimizing the Optimal Growth Conditions for Mercury-Tolerant Strains

**Figure 2:**
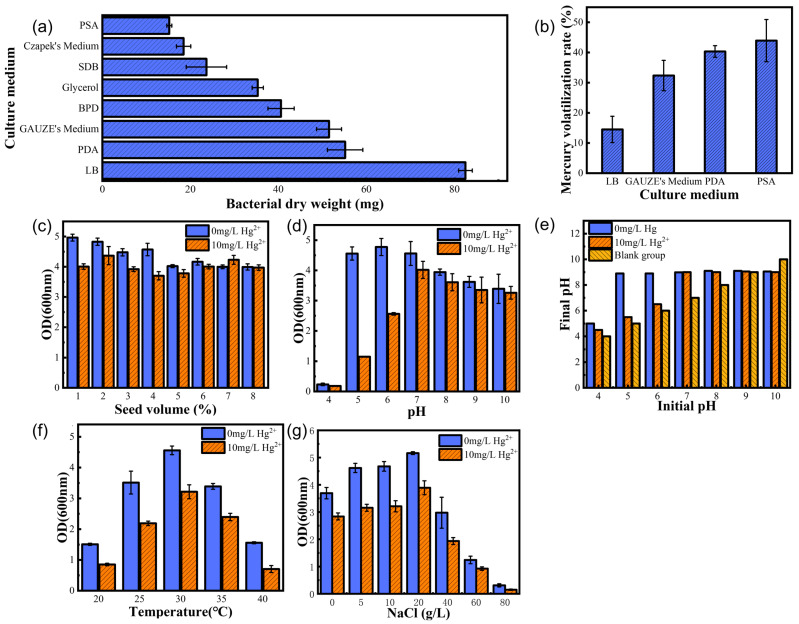
(**a**) Growth of the LBA119 strain in different media and (**b**) volatilization of mercury in different culture media; (**c**) effect of different strain inocula on the growth status of the LBA119 strain; (**d**) effect of pH on the growth of the LBA119 strain and (**e**) effect of the LBA119 strain on environmental pH; (**f**) effect of temperature on the growth of the LBA119 strain; (**g**) effect of salt concentration on the growth of the LBA119 strain.

A comparison of the growth of LBA119 in different media is shown in Figure 2a. The experimental results showed that the magnitude of growth of the LBA119 strain in different media (10 mg/L Hg^2+^) was LB Culture (LB) > Potato Dextrose Agar (PDA) > Czapek Dox Medium (Czapek’s Medium)> Beef extract peptone medium (BPD) > Glycerol Medium (Glycerol) > Sabouraud Dextrose Broth Medium (SDB) > Gauze’s Synthetic Broth Medium (GAUZE’s Medium)> Potato Saccharose Agar (PSA).

The volatilization of mercury in different media is shown in Figure 2b. The experimental results showed that, after 48 h of shaking incubation, the magnitude of mercury volatilization in the medium was in the following order: LB > GAUZE’s Medium > PDA > PSA.

The effects of different strain inocula on the growth status of the LBA119 strain are shown in Figure 2c. The biomass of this strain reached a maximum at 1% inoculum when incubated for 36 h without mercury. In the mercury-containing case, the inoculum level had less effect on the growth status of the strain, and the maximum biomass was reached at 2% inoculum, with a small increase in the growth of the strain when the inoculum level was increased to 7%. In the subsequent experiments with mercury addition, the optimal inoculum for this strain should be 2%.

The effect of the initial pH of the medium on the growth of the LBA119 strain is shown in Figure 2d. The experimental results showed that the LBA119 strain was adapted to weakly acidic, neutral, and alkaline environments but not to acidic solid environments, and its optimum growth pH was 6.0 in a mercury-free environment; its biomass (OD_600nm_) in a 10 mg/L Hg^2+^ environment was reduced compared with that in a mercury-free state, and its optimum growth pH was 7.0.

The effect of the LBA119 strain on the pH of the medium is shown in Figure 2e. When the initial pH value was 5.0–10.0, the pH value of the medium was stable in an 8.9–9.1 range after 36 h of incubation, which indicates that the microbial metabolic activity of the mercury-resistant strain changes the pH value of the environmental medium, and the LBA119 strain causes the pH value of the medium increase (or decrease) to be approximately 9.

As shown in Figure 2f, temperature causes a significant effect on the growth of the strain. The LBA119 strain exhibits a good growth rate from 25 to 35 °C, and its optimal growth temperature is 30 °C in mercury-containing and nonmercury-containing environments. Growth decreases significantly when the temperature falls below 20 °C and reaches 40 °C, so 30 °C is the optimal growth temperature for this strain.

A comparison of the growth of the LBA119 strain with varying salt concentrations is shown in Figure 2g. Based on the experimental results, without mercury, the LBA119 strain can grow in an LB medium containing 0–60 g/L NaCl; its biomass increases as the salt concentration increases, and its biomass reaches its maximum biomass at a salt concentration of 20 g/L. In an environment with 10 mg/L Hg^2+^, the strain can grow in 0–60 g/L NaCl, and its optimal salt concentration is 20 g/L. As a result, in mercury-containing environments, 20 g/L salt is the most appropriate salt level for the LBA119 strain.

### 3.5. Effect of Mercury Concentration on the Growth Status of the Strain

**Figure 3:**
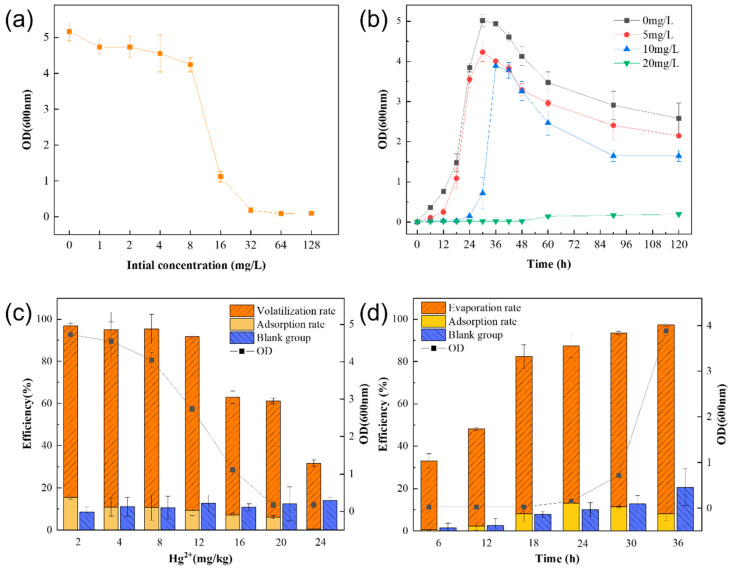
(**a**) Minimum inhibitory mercury concentration of the LBA119 strain and (**b**) growth curves at different mercury concentrations; (**c**) removal efficiency of LBA119 for different mercury concentrations; (**d**) effect of time on the efficiency of LBA119 for mercury removal.

The minimum inhibitory mercury concentration of strain LBA119 is shown in Figure 3a. The growth of strain LBA119 decreased with increasing heavy metal mercury concentration, and the growth of this strain in the LB liquid medium with 1, 2, 4, 8, 16, and 32 mg/L Hg^2+^ decreased by 11.24%, 8.30%, 11.75%, 25.48%, 78.39%, and 96.57%, respectively. The growth of the LBA119 strain almost stopped at Hg^2+^ concentrations greater than 32 mg/L. Therefore, the minimum inhibitory concentration (MIC) of Hg^2+^ for the LBA119 strain was 32 mg/L.

The growth curves of the LBA119 strain are presented under different mercury concentrations in Figure 3b. The growth pattern of LBA119 in the blank control was 0–30 h for the logarithmic growth period, and the OD_600nm_ started to decline after 36 h for the decay period. The OD_600nm_ of LBA119 at a low concentration (5 mg/L) of mercury decreased by 14.82%; when the mercury content was 15 mg/L, the growth of the LBA119 strain showed a delayed period (24 h), and its OD_600nm_ maximum value appeared at 36 h, which decreased by 44.81% compared with the control, but the overall growth curve shape was similar to the control growth curve. At a mercury concentration of 20 mg/L, the growth was severely inhibited, and its OD_600nm_ maximum value decreased by 96.09% compared with the control.

### 3.6. Mercury Volatilization and Adsorption Efficiency of the Strains

The volatilization and adsorption efficiency of the LBA119 strain with different mercury concentrations is shown in Figure 3c. At mercury concentrations of 2–12 mg/L, the mercury removal rate by this strain reached more than 90% and up to 96.89%. However, as the mercury concentration increased, the mercury removal rate decreased rapidly (from 65.6% at 16 mg/L to less than 32.28% at 24 mg/L).

Figure 3d shows the results of inoculating fresh bacterial solution in an LB liquid medium containing 10 mg/L Hg^2+^ and investigating the effect of incubation time on the mercury removal efficiency of the LBA119 strain. The experimental results indicated that the efficiency of mercury removal by the strain increased as time progressed, and the rate of mercury volatilization was significantly greater than the rate of mercury adsorption. The total removal rate of the LBA119 strain increased significantly after 12 h and reached a maximum at 36 h. The rates of total removal, volatilization, and adsorption were 97.32%, 89.08%, and 8.24%, respectively.

### 3.7. Fourier Transform Infrared Spectroscopy Studies-Adsorption Mechanism

**Figure 4:**
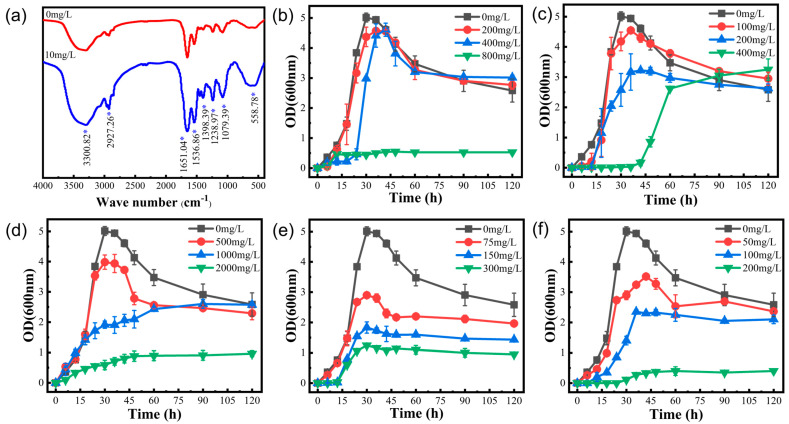
(**a**) FTIR spectra of the LBA 119 strain (* indicates peak increase). (**b**) Growth curves of LBA119 at different Mn^2+^ concentrations. (**c**) Growth curves of LBA119 at different Zn^2+^ concentrations. (**d**) Growth curves of LBA119 at different Pd^2+^ concentrations. (**e**) Growth curves of LBA119 at different Cd^2+^ concentrations. (**f**) Growth curves of LBA119 at different Cr^6+^ concentrations.

To investigate the mechanism by which strain LBA119 responds to Hg stress, changes in the adsorption functional groups of bacteria before and after treatment with the heavy metal Hg^2+^ were analyzed by Fourier transform infrared (FTIR) spectroscopy [21]. Figure 4a showed that the strain showed a significant increase in absorption peaks at 3300.82, 2927.26, 1651.04, 1536.86, 1398.39, 1238.97, 1079.39, and 558.78 cm^−1^ when the environmental Hg^2+^ concentration was 10 mg/L. The spectral peak at 3300.82 cm^−1^ is the stretching vibration of the OH from water bound by the stretching of the NH group from CH in the cell wall; the spectrum peak at 2927.26 cm^−1^ is CH3 of fatty acid; the peaks at 1651.04 cm^−1^ and 1536.86 cm^−1^ are the characteristic spectral bands of the protein; and the intense absorption peak at 1651.04 cm^−1^ is mainly caused by the stretching vibration of C=O. The strong absorption peak at 1536.86 cm^−1^ is primarily driven by the bending beat of NH, which are from the amide I (C=O) and amide II (NH) bands of the protein and peptide, respectively. The results indicate that the proteins amide I and amide II of the bacterial cell wall are actively involved in the adsorption process of heavy metal Hg^2+^. The peak at 1398.39 cm^−1^ is the stretching vibration of COOH. The peak at 1238.97 cm^−1^ is the stretching vibration of P-O and C-S and the superimposed absorption peak of C-O and C-OH. The peak at 1079.39 cm^−1^ is the stretching vibrational peaks of C-O-C and C-O-P, and 558.78 cm^−1^ is the vibrational absorption peaks of M-O and O-M-O (M is the metal ion), in which the absorption peaks are enhanced. Furthermore, the functional group may provide lone pairs of electrons to ligand-bind to Hg^2+^, indicating that, for the LBA119 strain under heavy metal Hg stress, Hg-O and O-Hg-O uptake levels were increased.

### 3.8. Effectiveness of Removing Different Heavy Metals

The results of the maximum tolerance concentrations of the LBA119 strain to a single heavy metal, as shown in Table 3, indicate that the LBA119 strain is highly resistant to heavy metal ions other than Hg^2+^. The strain showed extremely high resistance to Pb^2+^, Mn^2+^, Zn^2+^, Cd^2+^, and Cr^6+^ and weak resistance to Co^2+^ and Ag^+^. The tolerance concentration of LBA119 bacteria to heavy metal ions was Pb^2+^ > Mn^2+^ > Zn^2+^ > Cd^2+^ > Cr^6+^ > Hg^2+^. At low concentrations, heavy metal ions did not significantly affect the growth of the 119 strain. Consequently, this strain is heavy metal-resistant with remediation potential in heavy metal-contaminated environments.

The effects of other heavy metals on the growth curve of LBA119 are shown in Figure 4. The experimental results indicated that the growth of the strain was severely inhibited at the maximum tolerated concentration of heavy metal ions. The OD_600nm_ of the LBA119 strain reached its maximum at 30 h in the absence of heavy metals; the maximum peaks of Mn^2+^ (200 mg/L), Zn^2+^ (100–200 mg/L), Pb^2+^ (500 mg/L) and Cd^2+^ (75–300 mg/L) all occurred at 36 h. Under Zn^2+^ (400 mg/L) and Cr^6+^ (200 mg/L) environments, the lag period at the beginning of the growth of the LBA119 strain was prolonged, but the shape of the overall growth curve was similar to that of the control. Compared to that of the control, the OD_600nm_ maximum values of this strain were reduced by 89.10%, 35.19%, 75.26%, 82.15%, and 92.02% at the highest concentrations of Mn^2+^, Zn^2+^, Pb^2+^, Cd^2+^, and Cr^6+^, respectively, indicating that the growth of the LBA119 strain was substantially inhibited at the maximum tolerated concentration.

### 3.9. Remediation Effect of the LBA119 Strain on Mercury-Contaminated Soil

**Figure 5:**
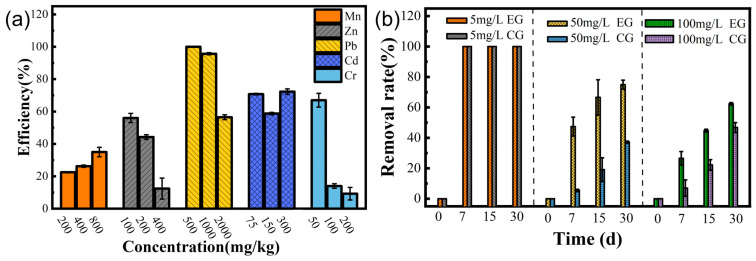
(**a**) Heavy metal removal rate of the LBA119 strain; (**b**) Bioremediation effect of the LBA119 strain on mercury-contaminated soil.

The effects of performing bioremediation with mercury-contaminated soil using the LBA119 strain was analyzed, as presented in Figure 5b. When the initial mercury concentration in the soil was 5 mg/L, both the remediation group inoculated with LBA119 and the control group inoculated with the LB medium without bacterial biomass reached a 100% removal rate after 7 days [22]. When the baseline Hg concentration was 50 mg/L, the total Hg removal rate was significantly increased by inoculation with LBA119, from 37.23% to 74.90% at 30 days, compared to that of the Hg-contaminated soil with an LB medium without bacterial biomass. When the initial mercury concentrations were 50 and 100 mg/L, the total mercury removal rate of the remediation group was higher than that of the control group. This value increased from 37.23% to 74.9% and from 46.81% to 62.35% after 30 days, respectively.

## 4. Discussion

Four factors affect the growth and reproduction of the strain: optimum medium, optimum temperature, optimum pH, and optimum inoculum. Firstly, the mercury experiments in different media showed (Figure 2b) that the volatility of mercury was higher in PSA, PDA, and GAUZE’s medium, probably because the media contained reducing sugars that could reduce Hg^2+^ and volatilize after reducing Hg^2+^ to Hg^0^. Therefore, these media are not suitable for studies related to the study of strain tolerance to Hg. Taner Sar et al. [23] studied the effect of inoculum level on ethanol production. We found that a low inoculum level resulted in a prolonged delay period. In contrast, a high inoculum level resulted in violent competition for survival among microorganisms and the death of a significant number of microorganisms. As shown in Figure 2d, too high or too low pH environments resulted in significant differences in strain growth, which is because extreme pH not only affects the activity and stability of enzymes in bacteria but also reduces microbial resistance to high temperatures and nutrient uptake, and also affects the ability of bacteria to produce fatty acids, which is consistent with the extremophile green alga Coccomyxa melkonianii at different pH values, which is consistent with the lipid production and morphological behavior of the extremophile green alga [24]. Since microorganisms do not possess thermoregulatory mechanisms, external temperature directly affects the activity of microbial intracellular enzymes, which are reduced at low temperatures. The metabolic rate and the ability of bacteria to synthesize substances decrease at low temperatures, leading to a decrease in reproductive capacity (Figure 2f), in agreement with the view of Rosa Margesin et al. [25], who studied the effect of different temperatures on a series of growth parameters of three bacteria. As shown in Figure 2c, in the mercury-containing environment, the strains showed easy protein secretion and expression under optimal growth conditions compared to the control, which would increase tolerance to inorganic mercury. As a result, we determined that the optimal growth conditions for this strain in a mercury-containing environment were an LB medium with 2% inoculum, 30 °C, and an initial pH of 7.0.

The logarithmic growth period of strain LBA119 was continuously delayed with increasing mercury concentration (Figure 3b). The growth and removal of mercury by this strain decreased with increasing mercury concentration (Figure 3a,c). However, mercury removal continuously increased with time (Figure 3d). Mercuric chloride is an inorganic mercury agent; the higher the mercury concentration, the more toxic it is. High mercury concentrations inhibit bacterial strains’ growth and their bacterial sulfhydrylase activity, causing impairment of bacterial metabolism. At mercury concentrations up to 32 mg/L, the LBA119 strain stopped growing because mercury ion reductase was inhibited at high mercury concentrations, the same as the mercury tolerance of Halomonas zincidurans [26].

As shown in Figure 4a, by comparing the IR spectra of strain LBA119 in the LB medium containing mercury (10 mg/L) and that without mercury, it was found that OH, -CH3, C=O in the amide I band, NH in the amide II band, P-O, C-S C-O, C-OH, -COOH, C-O-C, C-O-P, M-O, and O-M-O groups were enhanced. Amide I and amide II are characteristic absorption peaks of proteins. From these peaks, it can be assumed that proteins are involved in the adsorption process to the heavy metal Hg^2+^. The positively charged mercury ions combined with protein molecules to form protein salts, and irreversible precipitation of proteins (heavy metal ions precipitated proteins) occurred. The relationship between protein content and heavy metals indicated that specific proteins were critical for binding heavy metals [27]. The presence of excessive mercury concentrations may inhibit strain growth or even result in strain death.

The main mechanisms of bacterial resistance to heavy metals are biosorption, extracellular precipitation, biotransformation, bioaccumulation, and efflux. Table 2 and Figure 4b–f show the extensive resistance of strain LBA119 to other heavy metals, with severe inhibition of growth at the maximum tolerated concentration of heavy metal ions, in agreement with the previously discussed results.

Biosorption depends on many factors, such as surface adsorption, ion exchange, ligand, chelation, and micro-precipitation, as well as the type of functional groups of the bacterial biomass. As shown in Figure 5a, the strain showed some removal of different heavy metals, with the most effective removal of Pb. According to Hoyle-Gardner, J. et al. [28], at neutral pH, the electrostatic attraction between Pb ions and receptors on the bacterial cell wall might facilitate the adsorption of Pb by the strain, and Pb precipitates at weak acidic pH 2 and alkaline pH above 7, in the absence of bacteria, which is consistent with our results. The LBA119 strain adjusted the environmental pH (Figure 1e), and the mechanism of lead removal mainly relied on adsorption and the adjustment of environmental pH to precipitate lead ions.

Microbial soil remediation technology mainly relies on adsorption and transformation to remediate the soil and also has the characteristics of low investment, low pollution, and environmental friendliness. In the Orbetello Lagoon, Italy, Milva Pepi et al. [11] have isolated mercury-tolerant bacteria that can volatilize both organic and inorganic mercury into elemental mercury and contribute to mercury removal from sediment leachate as in the removal of mercury by the strain LBA119 we studied. Thus, LBA119 is resistant to mercury and can volatilize the ionic form of mercury metal (Hg^2+^) into its elemental form (Hg^0^). As shown in Figure 5b, inoculation of the LBA119 strain in high-concentration mercury-contaminated soil could improve the mercury removal rate compared with the control group, thus reducing the heavy metal mercury content in the soil. In conclusion, strain LBA119 has potential applications in the bioremediation of mercury-contaminated soils.

## 5. Conclusions

Heavy metal-contaminated soils from agricultural fields in mining areas may be an essential source for the isolation of many potentially heavy metal-tolerant bacteria. The mercury-tolerant bacterial strain LBA119 isolated from Luanchuan, Luoyang: (1) was identified as *Bacillus* by Gram staining, electron microscopy analysis, physiological and biochemical tests, and 16S rDNA identification; (2) the optimal growth conditions of the strain in the absence and presence of mercury were explored, and the growth, as well as the enzymatic activity of the strain, could be promoted under optimal conditions; (3) the strain was tested for minimum mercury inhibition concentration under optimum growth conditions, showing that the strain is highly tolerant of mercury and is capable of surviving in mercury-rich environments; (4) the bacteria isolated from the mine were found to remove mercury primarily by volatilization supplemented by adsorption, which can promote the volatilization of mercury in the soil and reduce the mercury content in the soil, emphasizing the significance of the strain to the area; (5) the bioremediation effect of isolated bacteria on mercury ranged from 62 to 97.36%, indicating that the strain is a promising candidate to be used for the bioremediation of heavy metals (especially Hg). This strain could be ideal for the microbial remediation of heavy metal-contaminated soil in mining areas.

## Figures and Tables

**Table 1:** Components of PCR reaction.

Reaction System	Volume (µL)	Reaction System	Volume (µL)
ddH_2_O	9.5	Primers 1492R	1
DNA Template	1	2 × Taq DNA Polymerase	12.5
Primers 27F	1	General System	25

**Table 2:** Physiological and biochemical characteristics of the LBA119 strain.

Identification Test	Test Results	Identification Test	Test Results
Starch hydrolysis	+	Methyl red test	+
Oil and grease hydrolysis	−	V-P	−
Glucose fermentation	+	Lactose fermentation	+
Indole test	+	H_2_S	−
Citrate utilization test	+	Motility experiments	+
Gelatin hydrolysis	+	Gram stain	+

Note: “+” indicates positive; “−” indicates negative.

**Table 3:** Tolerance of strain LBA119 to single heavy metals.

Heavy Metal Ions	MTC_G_ (mg/L)	MTC_Y_ (mg/L)
Mn^2+^(MnCl_2_·4H_2_O)	500	800
Zn^2+^(ZnSO_4_·7H_2_O)	180	400
Pb^2+^(PbC_4_H_6_O_4_·3H_2_O)	600	2000
Cd^2+^(CdCl_2_·5/2H_2_O)	300	300
Cr^6+^(K_2_Cr_2_O_7_)	200	200

## Data Availability

The authors declare that the data supporting the findings of this study are available within the article and its additional information files.
